# Extracting microplastic decay rates from field data

**DOI:** 10.1038/s41598-022-04912-w

**Published:** 2022-01-24

**Authors:** T. Metz, M. Koch, P. Lenz

**Affiliations:** Department of Physics, Philipps-Universität Marburg, Renthof 6, 35032 Marburg, Germany

**Keywords:** Environmental sciences, Ocean sciences, Physics

## Abstract

Being able to estimate and predict future microplastic distributions in the environment is one of the major challenges of the rapidly developing field of microplastic research. However, this task can only be achieved if our understanding of the decay of individual microplastic particles is significantly enhanced. Here, we show by using a rate equation model that currently available data of size distributions measured at single times cannot provide useful insights into this process. To analyze what data contains more information we generated more complex artificial data mimicking subsequent measurements using a stochastic simulation algorithm. Applying our model to this data revealed the following minimal requirements for future experimental data: (1) data should be collected as time series at identical spots and (2) size measurements should be combined with mass measurements. In contrast to currently available data, flux rates and decay parameters of individual particles can be extracted from such data.

## Introduction

Microplastic is commonly defined as plastic particles below five mm in size^1^. It can be divided into two groups, depending on its origin: primary microplastic is already produced in microscopic size, whereas secondary microplastic originates from the fragmentation of larger plastic items through chemical, physical or biological processes^2–4^. It is unclear which type of microplastic is more common in the ocean, but the secondary microplastic is assumed to dominate in the open sea^5,6^. It is undisputed, that all types of plastic eventually decay into smaller fragments with time^7^. However, so far little is known about the decay processes of microplastic particles in the environment. Open questions include the time scales of microplastic decay and its dependence on particle size, material, environmental conditions etc. In a recently developed modeling approach, the decay of buoyant macroplastic has been studied^8^ providing estimates for the decay rates of microplastic. Furthermore, the transport of microplastic has been included in hydrological models^9,10^. However, modeling had so far only a small impact on the field of microplastic research. Nevertheless, we are convinced and as we demonstrate in this study, modeling can provide important approaches to extract more information from field data. Furthermore, ways for standardized monitoring could be paved, that could significantly enhance the field of microplastic modeling. The goal of our approach is to analyze if useful information about the decay process of microplastic particles can be extracted from currently available data. With this in mind, this study works towards three broad-gauged tasks: First, we want to find possibilities to extract decay rates from field data. Second, we aim at creating models based on decay rates that have the potential to project microplastic particle numbers in the future. Third, we seek to identify guidelines for quantifying field data to provide sufficient information for the extraction of decay rates and modeling the fate of microplastic in the environment.

In our study we first extract decay parameters from single-time field data using a very simplified model that consists of a set of coupled differential equations. Those parameters are fed as decay rates into a simulation based on the Gillespie-algorithm. The Gillespie-algorithm is commonly used for the stochastic simulation of chemical reactions, especially in biochemistry. Here, we apply it to microplastic decay, where the decays are viewed as chemical reactions of first order. This simulation then aims at reconstructing information from field data, based on the identified decay parameters, starting from an initial particle distribution. Finally, we develop a more sophisticated generalized model that we use to extract decay rates from time-series field data and artificially generated time-series data. We use the information of these studies to develop guidelines, how empirical data can meet requirements for modeling purposes.

## Main

### Extraction of parameters from single time point field data

In most current studies, size distributions of microplastic particles are provided by using a discrete scheme for a single time and location. For example, in a study by Klein et al.^11^, observed particle sizes were represented using three bins for size ranges 0.63-5 mm (bin 1), 0.2-0.63 mm (bin 2) and 0.063-0.2 mm (bin 3). However, since time series are seldom available, field data does not yet reflect how such a size distribution changes with time.

To analyze which information about decay rates of microplastic can be extracted from single-time field data, we first set up a simple model consisting of three coupled differential equations and two decay rates. They describe the time evolution of the number of plastic particles in three size categories (bins). To simplify matters first, we made the following assumptions:

1. Neglect all particle characteristics apart from size.
2. There is no external influx or outflux of particles.
3. As the decay of particles in bin 3 is not further monitored we assume that they do not decay.
4. Upon decay, particles from bin 1 and 2 split up into two particles, both directly leaving their bin in order to go into the next bin.

The corresponding set of coupled differential equations is then given by

1 $$\begin{aligned} \begin{aligned} \frac{dn_{1}(t)}{dt}&= - \lambda _{1}n_{1}(t) ,\\ \frac{dn_{2}(t)}{dt}&= 2\lambda _{1}n_{1}(t) - \lambda _{2}n_{2} (t), \\ \frac{dn_{3}(t)}{dt}&= 2\lambda _{2}n_{2} (t). \end{aligned} \end{aligned}$$

Here $n_i(t),\ i=1,2,3$ denotes the number of particles in size category *i* and $\lambda _1$ and $\lambda _2$ are decay rates for particles in category 1 and 2, respectively.

While the model given by Eq. (1) does not provide an adequate description of microplastic decay in the environment, its purpose is to serve as starting point to find out which information can be extracted from field data. For instance, despite the simplifications already made, a discrete size distribution for one time point (which is a typical result of many publications) does not provide sufficient information to determine both $\lambda _1$ and $\lambda _2$ from the above equations. However, it allows the determination of a ratio ${\tilde{\lambda }} = \frac{\lambda _2}{\lambda _1}$ (for details and a derivation, see the methods section).

Because our approach is independent of size category boundaries, it is applicable to any size categorization and arbitrary size distributions. To illustrate this, we exemplary used the aforementioned field data from Klein et al. ^11^ and field data from Cabernard et al.^12^ which both consist of particle size distributions in three size categories to calculate values for the ratio of decay rates ${\tilde{\lambda }}$.

The results for the Klein et al. data (where particle numbers at ten different sites were reported) are shown in Table 1.

**Table 1:** Values of the ratio of decay parameters ${\tilde{\lambda }} = \frac{\lambda _2}{\lambda _1}$ as obtained from application of our decay model [see Eqs. (1) and (5)] to the field data of Klein et al.^11^.

Spot	M2	R7	R8	R6	R1	R5	R3	M1	R2	R4
${\tilde{\lambda }}$	**0.39**	**0.47**	**0.6**	**0.62**	**0.65**	**0.68**	*1.13*	*1.43*	*1.46*	*1.55*

Values are given in increasing order of ${\tilde{\lambda }}$. Values ${\tilde{\lambda }}<1$ are shown in bold, values ${\tilde{\lambda }} \ge 1$ are shown in italic. Spot location are denoted as in Klein et al.^11^: M1 and M2 are sites at the Main river which flows into the Rhine river between R1 and R2. R1-R8 are sites at the Rhine river. For both rivers larger numbers indicate a further downstream location.

**Figure 1:**
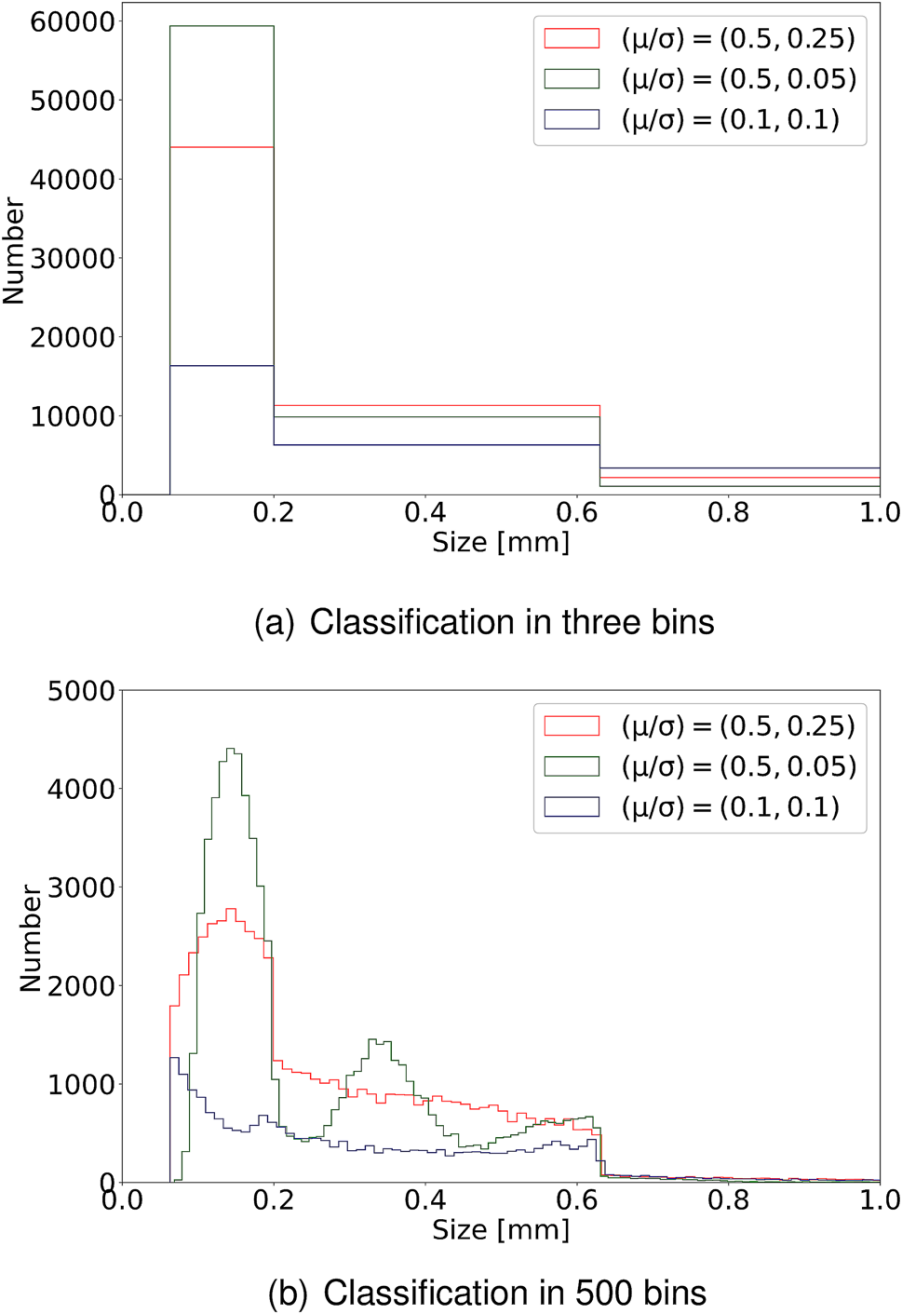
Size distributions projected into three bins (**a**) and 500 bins (**b**) as calculated from our simulations with ${\tilde{\lambda }} = 0.65$ and variations of $(\mu /\sigma )$. Asymmetric decays [for $(\mu ,\sigma )=(0.1,0.05)$, blue line] lead to a flatter and more uniform distribution. Symmetric decays with strong variations [for $(\mu ,\sigma )=(0.5,0.25)$, red line] show a high peak located at smaller particle sizes. Symmetric decays with little variation [$(\mu ,\sigma )=(0.5,0.05)$, green line] give the highest peak for small particles and show three distinct peaks of particle sizes.

As can be seen from Table 1, there is quite a variation between the different sites. From Fig. 1 of the publication of Klein et al.^11^ one sees that the sampling sites having a value of ${\tilde{\lambda }} > 1$ are in more densely populated areas, whereas those sites with ${\tilde{\lambda }}< 1$ are in more natural areas. Only M1 and M2 do not directly follow this trend.

The average ratio is ${\tilde{\lambda }} = 0.92 \pm 0.53$ with nearly the same number of sites with fractions below (6 sites) and above (4 sites) one. Thus, there is no indication that larger or smaller particles generally decay at significantly different rates in the investigated scenario.

In contrast, for the Cabernard et al. dataset, we obtain a value of ${\tilde{\lambda }} = 0.06$, which is significantly lower than all values obtained with the Klein et al. data. This value reflects a significantly higher value of $\lambda _1$ compared to $\lambda _2$, corresponding to a higher decay rate of particles in bin 1. Furthermore, according to Eq. (7) a low value of ${\tilde{\lambda }}$ corresponds to a higher value of $\tau$, which reflects that the given distribution formed later than the distributions of Klein et al.

Thus, the decay rates for the Cabernard et al. data show a much stronger size dependence than the Klein et al. data. In the case of Cabernard et al. large particles tend to decay faster than smaller particles. This is plausible as larger particles have a larger surface and are thus exposed to stronger physical forces. In the case of Klein et al., decay might rather be influenced by factors other than size, that so far have not been taken into account. A possible explanation for this difference is that the Klein et al. data was collected in sediment while the Cabernard et al. data was taken at the water surface, where particle decay is generally stronger and more likely to be influenced by size, due to sun exposure and other size related physical forces^13,14^. The observation that the Cabernard et al. microplastic distribution formed at a later point than the Klein et al. size distribution fits well to transport pathways of microplastic, where particles are typically transported from rivers to the open sea^3^.

Generally, however, the above analysis shows that even under the simplifying assumptions leading to our very idealistic model given by Eq. (1) single-time microplastic size distributions provide information that is only sufficient to extract a single parameter, the ratios of decay rates. In this example, there is only one relevant ratio for 3 sizes. It is easy to see, that in a similar way, data with *n* size bins determine $n-2$ ratios of size associated decay rates. As mentioned, the purpose of our simple model is to explore what information about decay rates can be extracted from field data. In our description we neglect many physical and chemical effects that actually occur in the environment. Such additional effects can be included in our description but require more detailed data in order to determine the additional parameters characterizing these effects. This point is elaborated further in the following subsections.

### Using the extracted parameters in a stochastic simulation algorithm

To analyze the influence of some of the simplifying assumptions underlying our model, we implemented a scheme to numerically solve Eqs. (1) [in its rescaled form (5)] based on the Gillespie-algorithm. We used the previously calculated values for ${\tilde{\lambda }}$ as decay rates in this simulation and analyzed how well it can reproduce particle distributions from the original field data from Klein et al.^11^. The main feature of this simulation is the possibility of tracking each particle, its decays and the size of the resulting particles during the time evolution of the system individually. In contrast to the model in the previous section, we included the more realistic possibility that particles resulting from a decay could stay in their initial size bin if the size after decay required it.

Implementation of the algorithm was done as follows: An initial number of particles with size 5 mm was set. We implemented two reactions: a split of particles in bin 1 and bin 2 with rate $\lambda _1 = 1$ and $\lambda _2 = {\tilde{\lambda }}$ (calculated for each of the ten sampling sites introduced in Sect. 2.1). The particle sizes after decay were drawn from a Gaussian distribution with mean $\mu$ and variance $\sigma$ (for details see the methods section). For each time step of the simulation, the fraction of particles in each of the three bins and the total number of particles $n_i/N_{tot}$ was calculated and compared to the fraction obtained from the field data of Klein et al.^11^. The simulation ran until a time of $\tau =6$. The minimal difference between simulation and field data, according to the following equation, that was reached within that time was noted as optimal fit

2 $$\begin{aligned} \Delta (\tau )_{opt} = \sqrt{\sum _{i=1}^3\left( \left( \frac{n_i(\tau )}{N_{tot}(\tau )}\right) _{\mathrm {sim}}-\left( \frac{n_i}{N_{tot}}\right) _{\mathrm {data}}\right) ^2}. \end{aligned}$$

Here, $n_i(\tau )$ and $N_{tot}(\tau )$ refer to the number of particles in bin *i* and the total number of particles at time step $\tau$, respectively. The indices ’sim’ and ’data’ distinguish between simulated and observed data.

Table 2 shows the obtained minimal value $\Delta =\Delta (\tau _{end})$ at which the simulation stopped. This value depends on the specific choice of $\mu$ and $\sigma$ as well as of ${\tilde{\lambda }}$, i.e.

$$\begin{aligned} \Delta =\Delta (\mu ,\sigma ,{\tilde{\lambda }}). \end{aligned}$$

As we are primarily interested in the dependence on ${\tilde{\lambda }}$ we chose three particular sets of $(\mu ,\sigma )$, two with $\mu = 0.5$ (preferring symmetric splits upon decay) and one with $\mu = 0.1$ (preferring strongly asymmetric splits upon decay).

**Table 2:** Values of $\Delta$ as obtained from Eq. (2) by minimizing the differences between numerically calculated size distributions of our decay model [see Eqs. (1) and (5)] and the field data of Ref.^11^.

Spot	M2	R7	R8	R6	R1	R5	R3	M1	R2	R4	${\overline{\Delta }}$
${\tilde{\lambda }}$	0.39	0.47	0.6	0.62	0.65	0.68	1.13	1.43	1.46	1.55	
$\Delta (\mu =0.5,\sigma =0.05,{\tilde{\lambda }})$	0.09	0.02	0.06	0.08	0.09	0.1	0.04	0.01	0.02	0.03	$0.053 \pm 0.03$
$\Delta (\mu =0.5,\sigma =0.25,{\tilde{\lambda }})$	0.12	0.04	0.09	0.1	0.12	0.13	0.05	0.01	0.04	0.04	$0.074 \pm 0.04$
$\Delta (\mu =0.1, \sigma =0.05,{\tilde{\lambda }})$	0.09	0.03	0.04	0.05	0.07	0.07	0.02	0.02	0.05	0.03	$0.050 \pm 0.03$

Values are given in increasing order of ${\tilde{\lambda }}$. The Gillespie-algorithm was run for three different combinations of the parameters of the Gaussian distribution ( $\mu$, $\sigma$) governing the size of particles after decay.

Table 2 shows, that there is some variation between the minimal value $\Delta$ that is obtained for the different sites and decay variants. Values with a ${\tilde{\lambda }}>1$ seem to have lower values of $\Delta$, with the exception of R7, which has low values, too. The spatial average ${\overline{\Delta }}$ depends on $\mu$ and $\sigma$. Within range of standard deviations ${\overline{\Delta }}(\mu =0.5,\sigma =0.05) \simeq {\overline{\Delta }}(\mu =0.1,\sigma =0.05)$, while ${\overline{\Delta }}(\mu =0.5,\sigma =0.25)$ is larger than the other two values.

Figure 1 shows examples of size distributions of particles that minimize equation (2) for each decay variant. Data were produced using ${\tilde{\lambda }} = 0.65$ for all combinations of $\mu$ and $\sigma$. The data for this figure were produced using ${\tilde{\lambda }} = 0.65$ for all combinations of $\mu$ and $\sigma$. Figure 1a shows the projection into three bins. However, as we tracked the size of all particles specifically in the simulation we can provide much more details about the size distribution. As an example, Fig. 1b shows the final size distribution projected into 500 bins. As can be seen by direct comparison of the two figures, that in a typical experimental size measurements (using 3 bins), it is very difficult to discriminate between the different decay variants: Most particles end up in bin 3 and in bin 1 and bin 2, all decay variants have particle numbers in a quite similar range. Figure 1b allows a more profound distinction between the decay variants. There, parameter values favoring asymmetric decays [for $(\mu ,\sigma )=(0.1,0.05)$, blue line] give rise to a rather flat distribution having the lowest number of particles. Values favoring symmetric decays with strong variations [for $(\mu ,\sigma )=(0.5,0.25)$, red line] give rise to a distribution showing a single peak located at smaller particle sizes. Only for parameter values favoring symmetric decays with little variation [$(\mu ,\sigma )=(0.5,0.05)$, green line] three distinct peaks of particle sizes are found.

In conclusion, all used decay variants achieve a quite similar ratio between particles in each of the three chosen bins and the total amount of particles (which was the target parameter). The simulation method is thus able to reproduce the target parameter from field data using the previously calculated decay parameter ${\tilde{\lambda }}$ to a reasonable extent. However, in all three decay scenarios we obtain different particle size distributions despite having comparable values in the cost-function [see Eq. (2)]. Thus, the details how particles decay apparently play an important role for the exact size distribution. But the size distributions projected into three bins does not provide enough information to discriminate well between the cases. This indicates, that field data quantified with a higher amount of size categories could be useful for our approach, in order to have more constraints on the microplastic distribution. This, of course, would also lead to a more sophisticated set of differential equations than those in equation (1), incorporating a higher amount of bins and decay rates.

At last, influx of particles might have a strong effect on the time evolution of size distributions. So far, this effect is not taken into account in our description. However, it is clear that inclusion of influx terms in our model (1) leads to three additional parameters, whose values cannot be determined from the comparison with the field data. In Sect. 2.3 we introduce a generalized approach that allows us to determine these influx parameters.

### Extraction of parameters from time-dependent size distributions

The previous section showed, that we are able to model the decay of microplastic using a Gillespie-algorithm, and to reconstruct field data information under simplified conditions. However, the results were obtained with simplified models which neglected influxes and outfluxes, which is most likely not legitimate in most instances.

As we will show here, much more information can be extracted from size distributions if instead of a single time snapshot, a time series of size distributions is measured. First, we analyze time series data from Zhao et al.^15^, who quantified microplastic particles at three different times (hereafter referred to as $t_0, t_1, t_2$) for three size categories. We furthermore analyze artificial data that we generate here using a Gillespie-algorithm. For data analysis, we use a model of higher complexity than in the previous sections, which represents one further step towards more realistic scenarios. The model is given by the following set of differential equations

3 $$\begin{aligned} \begin{aligned} \frac{dn_{1}(\tau )}{d\tau }&=(\lambda _{11}-\lambda _{22}-\lambda _{23})n_{1} (\tau ) +j_{1} \\ \frac{dn_{2}(\tau )}{d\tau }&=(\lambda _{12} + 2\lambda _{22} + \lambda _{23})n_{1}(\tau )+ (\lambda _{22}-\lambda _{33})n_{2} (\tau ) + j_{2} \\ \frac{dn_{3}(\tau )}{d\tau }&=(\lambda _{13} + \lambda _{23})n_{1}(\tau ) + (\lambda _{23}+2\lambda _{33})n_{2} (\tau ) + j_{3}. \end{aligned} \end{aligned}$$

Here, $n_i(\tau )$, $i=1,2,3$ are the number of particles in bin *i* at time $\tau$. $\lambda _{ij}$ are the decay rates, where *i* and *j* are the bins of new particles after decay. $j_i$ quantify the influx and outflux rates (positive $j_i$: influx, negative $j_i$: outflux). We explicitly assume here, that decay is only dependent on size. While this is a strong simplification, this type of model can in the future be easily adapted to incorporate other decay-influencing parameters by introducing more rates.

#### Analyzing field data

The equations (3) were solved by using the observed particle size distribution $n_i(0)$ at time $t_0$ as initial condition. The solutions were fitted to the particle numbers of the field data at $t_0$, $t_1$ and $t_2$ by minimizing the following equation with the $j_i$ and $\lambda _{ij}$ as optimization parameters

4 $$\begin{aligned} \Delta ^2 = \sum _{i=1}^{3}\sum _{\tau =0}^{2} (n_i(\tau )_{\mathrm {data}}-n_i(\tau )_{\mathrm {mod}})^2. \end{aligned}$$

Here $n_i(\tau )_k$ refers to the number of particles in bin *i* at time $\tau$ in the field data (*k*=data) and as calculated from the solution of model (3) (*k*=mod). Due to the large difference between the $t_i$ (several months) one expects significant differences in the flux rates during the observation frames. To account for this, we assumed that in the individual observation frames the effect of the flux can effectively be taken into account by replacing it by a constant value representing its average value in the time frame. In this way, the flux can vary between the different time frames yielding an approximation to its full time dependence.

Tables 3 and 4 show the values of the model parameters and $\Delta ^2$ obtained by the optimization procedure. The average flux rates in the time frame between $t_0$ and $t_1$ is denoted by $j_{i,1}$ (for bin *i*), and by $j_{i,2}$ for the time frame between $t_1$ and $t_2$ (and bin *i*).

**Table 3:** Estimated flux rates after minimization.

$\Delta ^2$	$j_{1,1}$	$j_{2,1}$	$j_{3,1}$	$j_{1,2}$	$j_{2,2}$	$j_{3,2}$
0.0005	$-$ 41.8	104.5	112	164.3	260	214.9

**Table 4:** Estimated decay rates after minimization.

$\lambda _{11}$	$\lambda _{12}$	$\lambda _{13}$	$\lambda _{22}$	$\lambda _{23}$	$\lambda _{33}$
0.01	0.003	0.0004	0.003	0	0

The low value of $\Delta ^2$ indicates very good correspondence between field data and model. However, the obtained flux rates differ significantly in the two time frames with different signs for bin 1 and values of $j_{2,2}$ and $j_{3,2}$ that are roughly twice as large as those of $j_{2,1}$ and $j_{3,2}$. This indicates that particle influx increased at later times. Furthermore, the influx rates are much larger than the decay rates showing that the time development is dominated by the particle in- and outflux. This is in accordance with the findings of Zhao et al., who assumed that particle numbers are dominated by river discharge^15^. Nevertheless, this demonstrates that our simple model can be used to extract relevant parameters from time series, although the time resolution is not sufficient to obtain sufficient information about the decay rates.

#### Analyzing artificial data

In a next step we wanted to find out which requirements field data has to fulfill to allow the extraction of meaningful information about the decay rates. To do this we generated artificial data that we then analyzed using our model given by Eqs. (3). Therefore, we implemented a numerical scheme based on the Gillespie-algorithm^16^ that proceeded as follows: we considered three size categories ($n_{1}$=number of particles with size 0.63 mm–5 mm, $n_{2}$=number of particles with size 0.2 mm–0.63 mm, and $n_{3}$=number of particles with size 0 mm–0.2 mm). Particles of all size categories can decay, resulting in two new particles. The size of the new particles is defined as a fraction of the selected initial particle’s size drawn from a Gaussian distribution (with mean $\mu$ and variance $\sigma$). New particles are distributed into bins 1-3 according to their size. Furthermore, we now allow for an influx and outflux of particles.

2000 particles with randomly distributed sizes between 0.63–5 mm served as starting conditions for the simulations. We used two different decay variants [Gaussian distribution for daughter particle size with $(\mu ,\sigma )= (0.5,0.25)$ and $(\mu ,\sigma )= (0.1,0.1)$]. All simulations run for a fixed amount of time, i.e. in units of simulation time until $\tau =3$. We kept track of the size distributions at time steps $\tau = 0,1,2,3$ to simulate field data taken at the same location over a period of time. A more detailed description of the implementation can be found in the methods section.

To test the influence of the influx, we minimized Eq. (4) (where the sum of time points now extends from $\tau =0$ to $\tau =3$) in two different ways: (1) by optimizing over all 9 parameters ($\lambda _{ij}$ and $j_i$), and (2) by optimizing over $\lambda _{ij}$ and fixing the influx rates $j_i=1000$ (at the values for which the data was generated).

Table 5 shows values of the model parameters and $\Delta ^2$ obtained by the optimization procedure.

**Table 5:** Estimated parameter values after minimization for artificial data.

$(\mu ,\sigma )$	$\Delta ^2$	$j_1$	$j_2$	$j_3$	$\lambda _{11}$	$\lambda _{12}$	$\lambda _{13}$	$\lambda _{22}$	$\lambda _{23}$	$\lambda _{33}$
Data	–	1000	1000	1000	0.1	0.1	0.1	0.1	0.01	0.1
(0.5,0.25) ($j_i$ given)	3.3	1000	1000	1000	0.09	0.11	0.11	0.1	0.001	0.1
(0.5,0.25)	3.9	1001	962	997	0.13	0.14	0.01	0.05	0.1	0.06
(0.1,0.1) ($j_i$ given)	2.5	1000	1000	1000	0.11	0.1	0.09	0.09	0.02	0.09
(0.1,0.1)	6.9	1002	1029	1067	0.15	0.06	0	0.07	0.08	0.07

**Figure 2:**
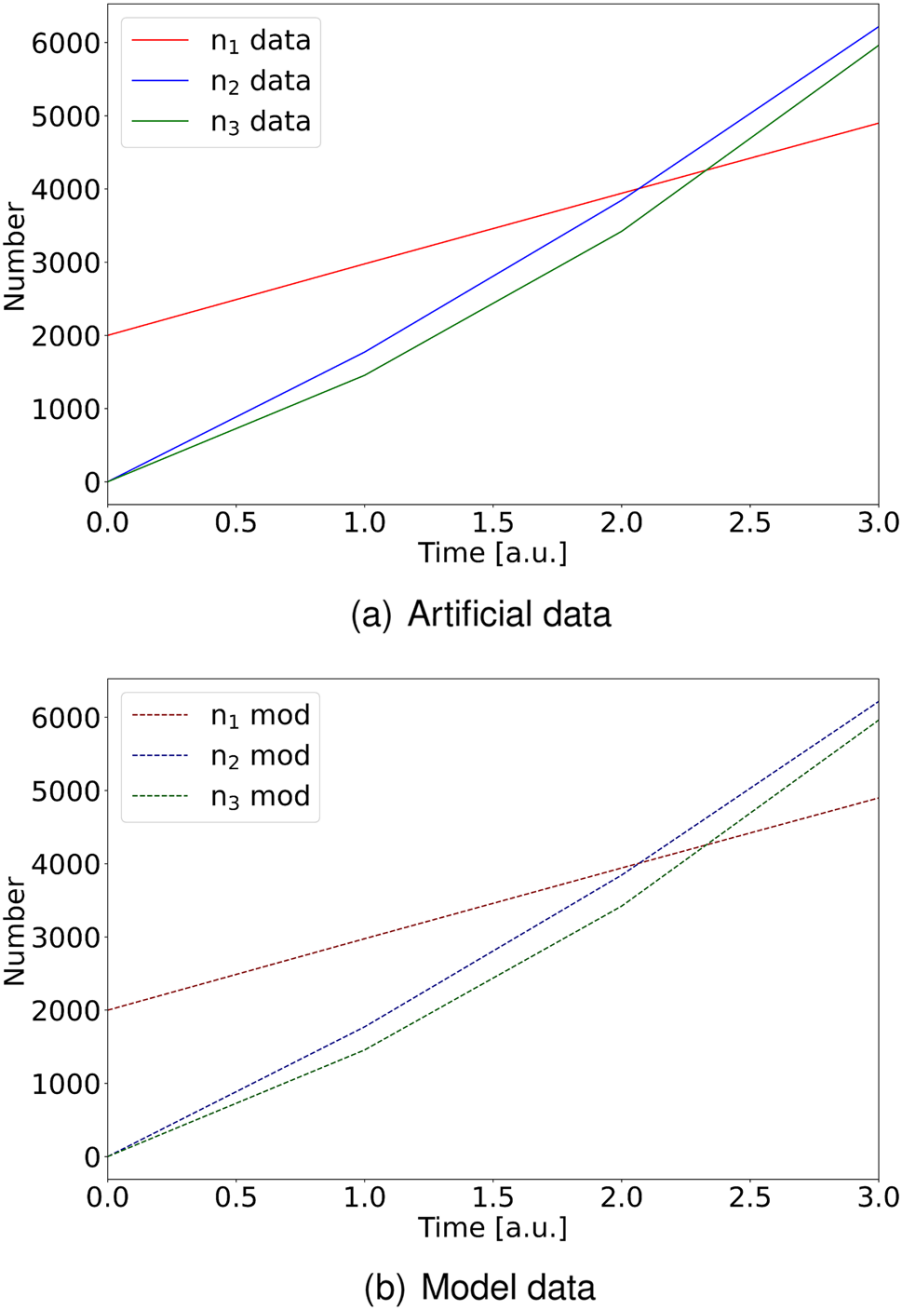
The figure shows the generated artificial data (**a**) using $\mu /\sigma = 0.1/0.1$ compared to the model data (**b**) over time in case that $j_i$ were not provided during optimization. As can be seen from Table 5, this case had the largest difference between artificial data and model. However, even in this case the curves representing data and model cannot be distinguished by direct inspection.

As can be seen from Table 5, agreement between prescribed and obtained values is significantly better if the values for the influx rates are provided. This is the case for both decay variants, and is also reflected in the value for $\Delta ^2$, which is slightly lower in these cases. Largest deviation between data and model occurs for the case $(\mu ,\sigma )=(0.1,0.1)$ with the influx rates $j_i$ subject to optimization. However, Fig. 2 shows, that even in this case the time dependence of the particle numbers in each bin is very well captured by the model. Data (full line) and model (dotted line) are indistinguishable. This is also the case for the other three scenarios, which lead to nearly identical plots.

From Table 5 and Fig. 2 it becomes furthermore apparent, that providing influx rates does not give significantly better results for $\Delta ^2$ (i.e. agreement between data and model curve). However, better correspondence of prescribed and optimized $\lambda _{ij}$ values is the key advantage. This is due to the fact, that for this limited amount of data it is very difficult to discriminate between particle inflow (with rate $j_i$) and a particle decay into two particles belonging to the same size bin (occurring with rate $\lambda _{ii}$). Thus, the influx parameters are critical variables whose values should be determined experimentally or a least be constrained, for optimal decay rate estimation.

One way to realize this, is to take into account the mass of the particles. On the model side this could be easily done but on the experimental side this requires a specific quantification method. While mass of particles is reported in some studies (e.g. Hurley et al.^17^), most studies only report particle counts in size categories. Furthermore, our model approach needs data from subsequent measurements reporting both particle numbers and particle weights for particles in size categories, as already proposed by Metz et al.^18^. This time series data would then include a size histogram with particle counts and weights. Any increase or decrease could then be associated with particle influx or outflux in order to constrain the influx rates $j_i$.

A drawback of our current decay model is that it so far only takes into account size as a particle characteristic. Other parameters, such as shape and composition, are neglected. However, those parameters can have a substantial effect on particle degradation as well. This is especially due to the fact that typical physical forces that lead to degradation, such as sunlight, are strongly influenced by particle shape and composition^13^. To allow for a more realistic representation of particle decay in the environment, those particle characteristics would need to be incorporated in our model equations, by setting up Eq. (3) separately for particles of different shape or composition. However, this would also make more detailed data necessary to allow for the estimation of unknown parameters.

This shows that the commonly chosen approach to quantify microplastic abundance by using single spot and single time size distributions has significant drawbacks when it comes to gaining information for modeling approaches. Currently available time series, such as in Zhao et al.^15^, Imhof et al.^19^, Zobkov et al.^20^ or Su et al.^21^ are a good step forward, but still have remaining issues to allow for modeling the decay of microplastic.

## Conclusion and outlook

In this work, we first worked with a very simple model for the decay of microplastic particles, that we used to show that it is impossible to extract all relevant decay parameters from single-time size distributions. This is only possible if more complex data is used. To demonstrate this, we used a generalized model incorporating multiple decay rates and which included influx and outflux of particles. We applied this model to time-series field data and artificially generated time-series data. We showed that the influx and outflux of particles has a significant influence on the extracted values of the decay parameters. In case of the artificial data, best agreement was found if the values of the flux parameters was provided. This is due to the fact that size distributions do not provide enough information to discriminate between inflowing particles and decays that result in particles with comparable size. Even our generalized model is still rather simple and neglects many processes. However, our findings justify its use as in many cases it is impossible to extract more detailed information from the experimental data. More complex models that involve more realistic descriptions of the underlying decay mechanisms have many more parameters that have to be determined making it even harder to extract useful information from experimental data.

Our theoretical findings support the following suggestions for an improved measurement protocol to obtain more meaningful data:

1. include particle mass as additional feature.
2. incorporate more size categories and correspondingly use additional size-related decay rates.
3. measure at spots with different influxes and outfluxes of particles.
4. use additional particle characteristics (such as shape, color, polymer composition etc.) that could potentially influence the particle decay and that provide additional information for the theoretical extraction of parameters.

For all samples, number of particles and mass abundance should be measured simultaneously for all particle categories (such as shape and composition) and reported in size categories. All data should be provided as time series. In this way it is guaranteed that the corresponding theoretical models can be solved and used to extract the relevant decay rates and influx and outflux parameters. These theoretical models can be obtained from our model given by Eq. (3) by generalizing it to the desired number of size bins and taking into account the additionally measured particle characteristics. Finally, it might be advantageous to select measurement spots according to expected influx and outflux of particles. For example, there might be spots where influx is much faster than decay, or spots where decay is much faster than influx. Given the results shown here, we expect that the latter spots are much easier to analyze.

## Methods

### Extracting decay parameters of microplastic from field data

Starting from Eq. (1) we introduce two new parameters

$$\begin{aligned} \tau&= \lambda _{1}t \\ \frac{\lambda _{2}}{\lambda _{1}}&= {\tilde{\lambda }}, \end{aligned}$$

where $\lambda _{1}$ and $\lambda _{2}$ are, respectively, decay rates for particles in bin 1 and bin 2. Eqs. (1) then become

5 $$\begin{aligned} \begin{aligned} \frac{dn_{1}(\tau )}{d\tau }&= - n_{1}(\tau ) \\ \frac{dn_{2}(\tau )}{d\tau }&= 2n_{1}(\tau ) - {\tilde{\lambda }}n_{2}(\tau ) \\ \frac{dn_{3}(\tau )}{d\tau }&= 2{\tilde{\lambda }}n_{2}(\tau ). \end{aligned} \end{aligned}$$

From these equations the interpretation of ${\tilde{\lambda }}$ becomes evident: If the ratio of decay rates ${\tilde{\lambda }}$ is $>1$ ($\lambda _{2} > \lambda _{1}$), particles in bin 2 decay faster than particles in bin 1 and if ${\tilde{\lambda }}$ is $<1$ ($\lambda _{1} > \lambda _{2}$), particles in bin 1 decay faster than particles in bin 2. However, in the absence of any external fluxes, depletion of bin 2 requires ${\tilde{\lambda }} >2$, as a particle of bin 1 decays into 2 particles of bin 2.

We solved these equations for initial conditions

$$\begin{aligned} n_1(0)=2000, n_2(0)=0, n_3(0)=0. \end{aligned}$$

As the solution is independent of the total number of particles, it is useful to introduce the ratios

6 $$\begin{aligned} \frac{n_{1}(\tau )}{n_{2}(\tau )} = a, \quad \frac{n_{2}(\tau )}{N_{tot}(\tau )} = b, \text{ where } N_{tot}(\tau ) = n_{1}(\tau ) + n_{2}(\tau ) + n_{3}(\tau ). \end{aligned}$$

Then, upon rearranging Eq. (6), we obtain a solution for $\tau$

7 $$\begin{aligned} \tau = \frac{\log (2) + \log (a) - \log (1+2a-{\tilde{\lambda }}) }{-1 + {\tilde{\lambda }}}. \end{aligned}$$

Inserting $\tau$ into Eq. (6) and rearranging then yields ${\tilde{\lambda }}$ as function of *a* and *b*. Since *a* and *b* are simply particle ratios that can be measured in the field, we extracted ten values for ten spots from the publication by Klein et al.^11^.

### A Gillespie-algorithm based on parameters derived from field data

Eqs. (5) are a coupled system of first order reactions that can be numerically investigated using the Gillespie algorithm^16^. We implemented this algorithm in Python v3.7 along the lines of Erban et al.^22^ and Gillespie^16^.

More specifically, we used the 3 size categories of Klein et al.^11^, i.e. $n_{1} =$ number of particles in the size range 0.63 mm–5 mm, $n_{2} =$ number of particles in the size range 0.2 mm–0.63 mm, and $n_{3} =$ number of particles in the size range 0.063 mm–0.2 mm. Particles < 0.063mm are not considered, i.e. are not tracked specifically. An initial number of particles $N_{init}=2000$ with size 5 mm was used.

We implemented two reactions: A decay of particles in bin 1 with rate $\lambda _1$ and a decay of particles in bin two with rate $\lambda _2$, with the propensity functions $\lambda _1n_1$ and $\lambda _2n_2$. We performed ten simulations with $\lambda _1=1$ and $\lambda _2 = {\tilde{\lambda }}$ (see Sect. 2.1). A particle decay resulted in two particles of different sizes: particle 1 has the size of the initial particle multiplied with *x*, particle 2 has the size of the initial particle multiplied with $1-x$. For each reaction, *x* was randomly chosen between 0 and 1 using a Gaussian distribution with mean value $\mu$ and variance $\sigma$. We varied $\mu$ between 0.5 and 0.1. For a value of $\mu = 0.5$, $\sigma$ was varied to be 0.25 or 0.05 For $\mu = 0.5$, $\sigma$ was 0.05.

An optimization routine was implemented, to minimize the differences between the calculated particle size distributions and the experimental data of Ref. ^11^. To do so, the fractions

$$\begin{aligned} \left( \frac{n_1}{N_{tot}}\right) _{\mathrm {data}}, \left( \frac{n_2}{N_{tot}} \right) _{\mathrm {data}}, \left( \frac{n_3}{N_{tot}}\right) _{\mathrm {data}}, \end{aligned}$$

were calculated from the data in the publication for each sample and compared with

$$\begin{aligned} \left( \frac{n_1(\tau )}{N_{tot}(\tau )} \right) _{\mathrm {sim}}, \left( \frac{n_2(\tau )}{N_{tot}(\tau )}\right) _{\mathrm {sim}}, \left( \frac{n_3(\tau )}{N_{tot}(\tau )}\right) _{\mathrm {sim}}, \end{aligned}$$

obtained at each time step of the simulations.

The algorithm ran until a time $\tau =6$ and the minimum difference between field data plastic distribution and simulated plastic distribution according to Eq. (2) was selected as optimal fit.

### Using the Gillespie-algorithm to create artificial data

To create artificial data, a Gillespie-algorithm was implemented in Python v3.7, along the lines of Erban et al.^22^ and Gillespie^16^. The following size categories for microplastic particles were used: $n_{1}$ (particle species A) = 0.63 mm–5 mm, $n_{2}$ (particle species B) = 0.2 mm–0.63 mm and $n_{3}$ (particle species C) $= 0$ mm–0.2 mm).

An initial number of particles $N_{init} =2000$ was randomly and uniformly distributed between 0.63–5 mm at starting time $\tau = 0$. 11 reactions were implemented with the corresponding propensity function $\alpha _i$:

8 $$\begin{aligned}&A \xrightarrow []{\lambda _{11}} A + A~\alpha _1= \lambda _{11}n_1\end{aligned}$$

9 $$\begin{aligned}&A \xrightarrow []{\lambda _{12}} A + B~~~\alpha _2= \lambda _{12}n_1\end{aligned}$$

10 $$\begin{aligned}&A \xrightarrow []{\lambda _{13}} A + C~~~\alpha _3= \lambda _{13}n_1\end{aligned}$$

11 $$\begin{aligned}&A \xrightarrow []{\lambda _{22}} B + B~~~\alpha _4= \lambda _{22}n_1\end{aligned}$$

12 $$\begin{aligned}&A \xrightarrow []{\lambda _{23}} B + C~~~\alpha _5= \lambda _{23}n_1\end{aligned}$$

13 $$\begin{aligned}&B \xrightarrow []{\lambda _{22}} B + B~~~\alpha _6= \lambda _{22}n_2\end{aligned}$$

14 $$\begin{aligned}&B \xrightarrow []{\lambda _{23}} B + C~~~\alpha _7= \lambda _{23}n_2\end{aligned}$$

15 $$\begin{aligned}&B \xrightarrow []{\lambda _{33}} C + C~~~\alpha _8= \lambda _{33}n_2\end{aligned}$$

16 $$\begin{aligned}&\emptyset \xrightarrow []{j_1} A~~~\alpha _9= j_1\end{aligned}$$

17 $$\begin{aligned}&\emptyset \xrightarrow []{j_2} B~~~\alpha _{10}= j_2\end{aligned}$$

18 $$\begin{aligned}&\emptyset \xrightarrow []{j_3} C~~~\alpha _{11}= j_3 \end{aligned}$$

where $n_1$, $n_2$ and $n_3$ are the number of particles in bin 1, 2 or 3 at time $\tau$, respectively. *A*, *B* and *C* correspond to a particle belonging to bin 1, 2 or 3, respectively. This means, for example, that reaction (8) represents a decay of a particle in bin 1, that decays into two particles that are again size-wise belonging to bin 1. The rates $\lambda _{ij}$ are defined in such a way that exactly one particle in bin 1, 2 or 3 decays according to one of the respective reactions given above. The rates $j_i$ represent the probability that exactly one particle of bin 1, 2 or 3 gets created with randomly selected size within the appropriate size boundaries. This should mimic an influx. The following values were used: $j_1 = 1000, j_2 = 1000, j_3 = 1000, \lambda _{11}=0.1, \lambda _{12}=0.1, \lambda _{13}=0.1, \lambda _{22}=0.1, \lambda _{23}=0.01, \lambda _{33}=0.1$.

The time after which a reaction occurs is given by:

19 $$\begin{aligned} \tau = \frac{1}{\alpha _0}ln\left( \frac{1}{r_1}\right) , \end{aligned}$$

where $\alpha _0$ is the sum of all propensity functions at time *t*

20 $$\begin{aligned} \alpha _0 = \sum _{i=1}^{q}\alpha _i(t). \end{aligned}$$

After a reaction has been chosen, a particle within the appropriate bin was randomly selected. This initial particle’s size was multiplied with *x* and $(1-x)$, where *x* was randomly drawn from a Gaussian distribution between 0 and 1. We used two different Gaussian distributions with mean and standard deviation $(\mu , \sigma )= (0.5,0.25)$ and $(\mu ,\sigma )= (0.1,0.1)$. In doing so, random numbers *x* an $(1-x)$ were only accepted if the resulting daughter particles respect the size constraints of the chosen reaction and ended up in the correct size bins. Each simulation was run until a simulation time of $\tau = 3$ was reached. The number of particles in each size category resulting from the simulated decays was noted at the time steps: $\tau = 0$, 1, 2, 3. The differential equations (3) were solved for the initial conditions $n_1(0)=20$, $n_2(0)=0$ and $n_3(0) = 0$ using Mathematica v11.

A global minimization routine was used to identify those values of $\lambda _{ij}$ and $j_i$ for which the difference between the solutions of Eqs. (3) and the size distributions obtained by the field data and the Gillespie-algorithm was minimal. To do so, Eq. (4) (see main text Sect. 2.3) was minimized using scipy.optimize v1.3.1 with the “basinhopping” global minimization algorithm and the “COBYLA” local minimization algorithm. The values $j_1, j_2$ and $j_3$ were constrained to lie between 500 and 1500 in case of the artificial data and between -1000 and 1000 in case of the field data. The values for $\lambda _{ij}$ were constrained to lie between 0 and 1. The number of iterations was set to 25 000. In case of analyzing the artificial data, the initial point was randomly set to be $j_1 = 825, j_2 = 918, j_3 = 963, \lambda _{11}=0.59, \lambda _{12}=0.72, \lambda _{13}=0.22, \lambda _{22}=0.95, \lambda _{23}=0.32, \lambda _{33}=0.21$ for $(\mu ,\sigma )=(0.1,0.1)$ and $j_1 = 801, j_2 = 813, j_3 = 839, \lambda _{11}=0.1, \lambda _{12}=0.6, \lambda _{13}=0.12, \lambda _{22}=0.96, \lambda _{23}=0.6, \lambda _{33}=0.3$ for $(\mu ,\sigma )=(0.5,0.25)$. For both situations, one run was done with $j_i$ subject to minimization as well, while during one run only $\lambda _{ij}$ were optimized, while $j_i$ was fixed to the correct value of 1 000.

## Data Availability

The computer code used in this work is available from the authors upon request.
